# Efficacy of Tamsulosin plus Tadalafil versus Tamsulosin as Medical Expulsive Therapy for Lower Ureteric Stones: A Randomized Controlled Trial

**DOI:** 10.1155/2020/4347598

**Published:** 2020-01-29

**Authors:** Diwas Gnyawali, Manish Man Pradhan, Prem Raj Sigdel, Purushottam Parajuli, Sampanna Chudal, Sujeet Poudyal, Suman Chapagain, Bhoj Raj Luitel, Pawan Raj Chalise, Uttam Sharma, Prem Raj Gyawali

**Affiliations:** Department of Urology and Kidney Transplant Surgery, Tribhuvan University Teaching Hospital, Institute of Medicine, Maharajgung, Kathmandu 44600, Nepal

## Abstract

**Introduction:**

Urolithiasis is one of the common disorder with which about 1/5^th^ is found in the ureter, of which 2/3^rd^ is seen in the lower ureter. Medical expulsive therapy is one of the routine modalities of treatment which uses various drugs acting on the ureter smooth muscle by different mechanism. We aim to compare the efficacy of combination vs. single drug.

**Methods:**

This randomized controlled trial was done in 176 consecutive patients over a period of six months (March 2019 to August 2019) in Department of Urology and Kidney Transplant Surgery, Tribhuvan University Teaching. Participants were divided into two groups (Group A, tamsulosin plus tadalafil, and Group B, tamsulosin) from computer-generated random numbers. Therapy was continued for a maximum of 3 weeks. Stone expulsion rate, time to stone expulsion, analgesic use, number of colic and emergency room visits for pain, early intervention, and adverse effects of drugs were recorded.

**Results:**

Among 176 patients who were enrolled in study, 7 were lost to follow-up, and 5 people required immediate intervention. There was a significant higher stone passage rate in group A than group B (64 vs. 50; *P*=0.025) and shorter expulsion time (1.66 vs. 2.32 weeks *P*=0.001) and less number of emergency room visits and colic episodes. No significant side effects were noted during study.

**Conclusion:**

Tamsulosin plus Tadalafil is more effective than tamsulosin with early passage of stone and decreased number of colic episodes and emergency visits without significant side effects for lower ureteric calculi of 5 mm to 10 mm.

## 1. Introduction

Urolithiasis is one of the most common disorders of the urinary tract with life-time prevalence of up to 15% with men affected three times more than women [1, 2]. Improved quality of life may also have increased its prevalence. A significant proportion, about 1/5^th^ of urinary tract stones, is found in the ureter, of which 2/3^rd^ is seen in the distal ureter [3]. Initially, a colicky pain of various grades presents with ureteric stone. This is one of the most common problems that compel a patient to an emergency room [4].

Today, medical expulsive therapy (MET) has become the most used modality of a treatment for urolithiasis. During this treatment, the ureter smooth muscle is treated via various drugs by different mechanisms. Blocking alpha-(*α*-) 1 adrenergic receptors, especially in the distal third decreases basal smooth muscle contraction and causes propulsive antegrade peristalsis helping stone expulsion [2, 5, 6]. By increasing the intraureteral pressure gradient around the stone, alpha-1 adrenergic receptor antagonists eject distal ureteral stones [7]. Tamsulosin has continuously shown a proven role in increasing the stone expulsion rate and decreasing expulsion time [7]. Significant pathological changes can occur when ureteric stones are impacted. This can cause inflammatory reaction with mucosal edema which could further worsen the ureteric obstruction, increasing the risk of impaction and retention [8]. However, selective alpha-1 blockers, such as tamsulosin and silodosin, have been the treatment of choice, with proven efficacy in multiple clinical trials [8–10].

Recently, a newer PDE5 inhibitor, tadalafil, has shown action on nitric oxide-cyclic guanosine monophosphate-signaling pathway of smooth muscles, resulting in increased levels of cyclic guanosine monophosphate, causing ureteric relaxation [2, 11–13]. Due to its smooth muscle relaxation mechanism, tadalafil has received US Food and Drug Administration approval to treat many urinary tract diseases. Therefore, the combination of tamsulosin and tadalafil drugs is aimed to facilitate stone passage by better ureteric relaxation and reducing intramural ureter pressure. Though there have been few similar studies using various combinations, comparing the efficacy of tamsulosin and tadalafil vs. tamsulosin are very few, and these studies have taken longer duration of treatment (4 to 6 weeks) [5, 14] which might have affected the outcome. Meta-analysis [15] which also recommends further research has taken consideration of only one drug PDE5 inhibitor or alpha blocker in most of its studies inclusion. So, with this study, we aim to find out if the combination therapy is better than tamsulosin alone.

## 2. Materials and Methods

The study was conducted in the Department of Urology and Kidney Transplant surgery at Tribhuvan University Teaching Hospital, over a period of 6 months (from 1^st^ March 2019 to 31^st^ August 2019). All patients with lower ureteric stone from 5 mm to 10 mm in size, diagnosed by CT Scan, Ultrasound (USG) abdomen/pelvis or X-ray KUB, were only included in the study. CT scan was not done in all patients due to financial reason. Patients with the presence of multiple ureteric stones, radiolucent stones, urinary tract infection, pregnancy, and pediatric population; patients with a history of ureteral surgery or previous endoscopic procedures; patients having ischemic heart disease, congestive cardiac failure, or complicated hypertension; patients requiring emergency intervention; and patients with raised creatinine were not included in the study.

Previously performed other studies [5, 13, 16] were taken as a reference for *P*1 and *P*2 values for sample size, and it was calculated by the following formula and *K* value from (Table 1); a power of 80% and a level of significance of 95% was used for the test. With 10% drop out rate, the sample size was calculated as 176. The formula is

(1) $$\begin{array}{c} N=K\frac{p_{1}\left(1-p_{1}\right)+p_{2}\left(1-p_{2}\right)}{{\left(p_{1}-p_{2}\right)}^{2}}, \end{array}$$

where *N* = sample size, *p*_1_ = successful passage in the tamsulosin and tadalafil group, and *p*_2_ = successful passage in the tamsulosin group.

Written informed consent was taken from all participants. Study methodology and protocol were approved from the Institutional Review Board of Institute of Medicine, Tribhuvan University (reference no. 359(6-11) E2/075/76). This trial was registered retrospectively in the University Hospital Medical Information Network (UMIN) (registration no UMIN000038125).

Detailed history and clinical examination and routine urine examination and/or urine culture, serum creatinine, digital X-ray KUB and/or USG abdomen and pelvis, and/or CT-KUB were carried out in all patients. The stone size was determined using largest dimension.

**Figure 1:**
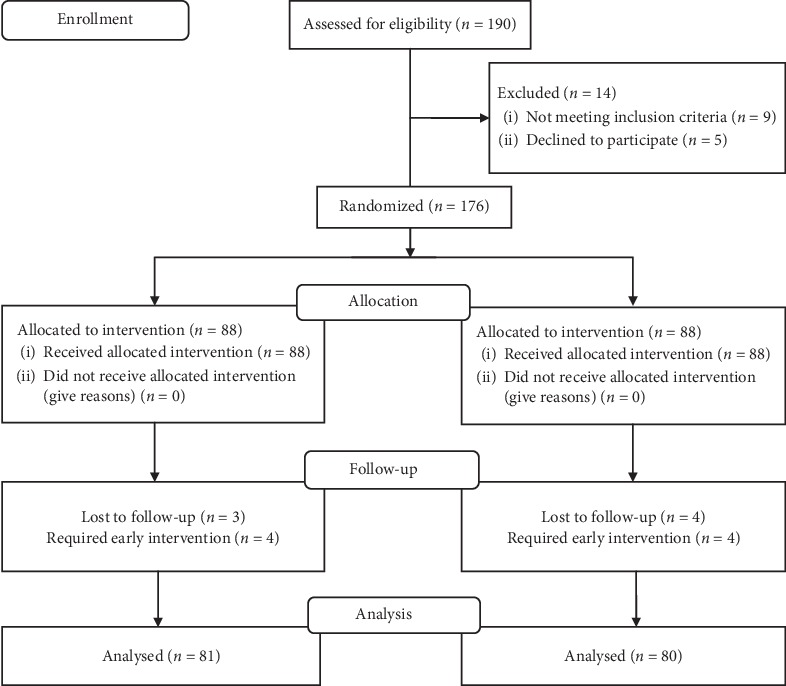
Patient flow diagram.

Patients were randomized and divided into two equal groups of 88 based on a computer-generated random number table (Figure 1). Patients in Group A were prescribed tamsulosin of 0.4 mg and tadalafil of 10 mg once a day, whereas those in group B were prescribed tamsulosin of 0.4 mg. Both groups received diclofenac of 50 mg three times a day, hyoscine butylbromide of 10 mg three times a day for one day followed by as per need basis, and proton pump inhibitor (Pantoprazole 40 mg) once a day. Drugs were continued until stone expulsion.

In both groups, drugs were continued until stone expulsion or for a period of 3 weeks. There was no strong evidence that, the longer duration of drugs user will increase the expulsion rate and deleterious effect of obstruction on kidney function will be minimized. Patients were instructed to take plenty of fluids and filter their urine by using a net. All patients were evaluated by physical examination, serum creatinine, and the same imaging modality by which lower ureteric stones were conformed previously in those who either could not present the stone or presented the stone that did not match the original size and shape. In case of doubt, CT KUB was done despite previous imaging modality to conform stone expulsion. Expulsion of the ureteric calculi, total dose of analgesic used, number of colic episodes and emergency room visits, and side effect of drugs were recorded. Semirigid ureteroscopy was done to those who did not pass stones after 3 weeks of follow-up for stone removal. Unpaired Student's *t*-test and the *χ*^2^-test were used for the analysis of the variables and categorical data. Differences were considered significant at a *P* value less than 0.05.

## 3. Results

Out of 190 patients, 176 met the inclusion criteria who were randomly assigned into 2 groups. Three patients from Group A and four patients from Group B lost their follow-up for various reasons. Four patients from both groups required early intervention, whereas the remaining patients completed the study. There were no statistically significant differences in patients' age, gender, and stone size (Table 2).

The stone expulsion rate was 79.0% in Group A and 62.5% in Group B; Group A showed a higher stone expulsion rate than Group B (*P* value = 0.025). The mean time for stone expulsion in Group A was 1.66 vs. 2.32 weeks in Group B (*P* value = 0.001). Out of 161 patients, stones were not expelled in 47 patients (17 and 30 patients in groups A and B, respectively) at the end of 3^rd^ week of therapy. These patients underwent semirigid ureteroscopic stone removal with laser lithotripsy. While comparing Group A (2.02), the patients had significantly less episodes of colicky pain than Group B (2.32) (*P* value = 0.001) with significantly less number of emergency room visits. Additionally, the mean requirement of analgesia (diclofenac) was significantly less in Group A (403) than in Group B (526) (Table 2).

Drug-related adverse effects such as headache, dizziness, postural hypotension, backache, and running nose were comparable between two groups (Table 3). Out of 58 males from Group A, 31 of them (55%) developed mild degree of penile tumescence lasting for 20–30 minutes, but none of them developed priapism.

## 4. Discussion

Urolithiasis is common urological disease. Among all urinary tract stones, about 1/5^th^ are ureteral stones, of which 2/3^rd^ are found in the lower third [17]. In last two decades, the development and improvisation of new, minimally invasive procedures (extracorporeal shock wave lithotripsy and ureteroscopy) for ureteral stones has considerably changed the management of ureteral stones [18, 19]. But on the contrary, these methods are not only risky and inconvenient but also very expensive. This can affect the quality of life of a patient as patient daily activities can be reduced [20]. Hence, there has been paradigm shift in the treatment of distal ureteric stone with a primary focus on MET. According to the available literature, spontaneous passage of distal ureteric stone using a conservative approach for stones of 5–10 mm is less likely [21], with a mean expulsion time of >10 days [20].

Factors influencing the spontaneous expulsion of stones, such as stone location, stone size, stone number, stone structure, ureteral spasm, mucosal edema or inflammation, and ureteral anatomy. Therefore, the use of medical therapy is justifiable to reduce edema, reduce spasm, and relax the smooth muscles for stone expulsion [21]. MET has recently emerged as an alternative strategy for the initial management of selected patients with distal ureteral stones [19, 20].

Since Sigala et al., described that the most common adrenoceptors found in the ureter are *α*-1D and *α*-1A [22], several studies have been carried out to determine the effect of a combined *α*-1A and *α*-1D selective antagonist, tamsulosin, which showed an improved expulsion rate of medium sized (3–10 mm) stones. We also observed an expulsion rate of 62.5% with tamsulosin, which is better than historical controls used in earlier studies of 30–43% [16, 23, 24].

Tadalafil, PDE-5 inhibitors, act via the nitric oxide/cGMP-signaling pathway, which results in increased levels of cGMP, leading to ureteric smooth muscle relaxation, which helps in stone passage [25, 26]. Tadalafil was used as it is more selective compared with sildenafil for PDE5 with long duration of action (∼36 h, and a half-life of 17.5 h) and its activity unaffected by meals [12] and combination of tamsulosin and tadalafil was found to be safe [27, 28] by Kloner et al. Bechara et al. showed effectiveness of combination when they used for LUTS [29].

While comparing the efficacy of drugs in our study, we found Group A (tamsulosin plus tadalafil) patients had higher expulsion rate than Group B (tamsulosin) 79.01% vs. 62.50% (*P* value 0.025), respectively. Stone passage rate in tamsulosin plus tadalafil was comparable with Jayant et al. of 83% [5] but was less than Rahman et al. of 90% [8] which may be they have used silodusin and tadalafil as combination.

Tamsulosin and tadalafil when used in combination facilitates stone passage and also decreases the stone passage duration 11.66 days which is shorter than 14.9 days of Jayant et al. [5] and comparable to 12 days of Rahman et al. [8].

In the present study, the mean analgesic requirement in group A was significantly less with respect to group B (403 mg vs. 531 mg (*P*=0.001)), this better pain control was also reflected by the lesser number of colic episodes and emergency room visits in group A. The abovementioned effects may be due to decrease in frequency and amplitude of phasic contractions that accompany ureteric obstruction; that is, an improved antispasmodic effect of tamsulosin and tadalafil [14].

There was no significant difference in side effects. These were mild and well tolerated by the study population who were relatively younger in age and lack of any comorbidity. Similar result was shown on various other studies. There was no significant difference in side effects between two groups and side effects were comparable with other studies [5, 8, 13, 14].

## 5. Conclusion

With this study, we can conclude that combination of tamsulosin and tadalafil is more efficacious than tamsulosin alone when used in lower ureteric stones of 5 mm to 10 mm with significant low-dose analgesic requirement, less number of colic episodes, and few number of emergency room visits without extra side effects.

## Figures and Tables

**Table 1:** *K* value for sample size calculation

Power				
*α*
		50%	80%	90%	95%
		*β* = 0.5	*β* = 0.2	*β* = 0.1	*β* = 0.05
	0.10	2.7	6.2	8.6	10.8
	0.05	3.8	7.9	10.5	13.0
	0.02	5.4	10.0	13.0	15.8
	0.01	6.6	11.7	14.9	17.8

**Table 2:** Demographic and results.

Parameter	Group A	Group B	*P* value
Mean age (years)	33.75 ± 10.01	32.85 ± 10.36	0.575
No. of patients (male/female)	58/23	50/30	0.243
Mean stone size (mm)	7.43 ± 1.23	7.34 ± 1.13	0.618
Expulsion rate (%)	64/81	50/80	0.025
Mean expulsion time (weeks)	1.66 ± 0.87	2.32 ± 0.76	0.001
Mean analgesic use (mg)	403 ± 131	526 ± 86	0.001
Mean no. of colic episodes	2.02 ± 0.80	2.34 ± 0.67	0.008
Mean no. of emergency visits	1.48 ± 0.61	1.70 ± 0.60	0.024

Statistical significance was analyzed by Student's *t*-test and *χ*^2^-test. Values are presented as mean ± standard deviation. Group A: tamsulosin and tadalafil. Group B: tamsulosin.

**Table 3:** Side effects.

Variable	Group A	Group B	*P* value
Headache	10	8	0.62
Dizziness	9	7	0.47
Postural hypotension	4	3	0.81
Backache	11	7	0.41
Runny nose	2	1	0.91
Abnormal ejaculation	5	6	0.78

Statistical significance was analyzed by the *χ*^2^-test. Group A: tamsulosin and tadalafil. Group B: tamsulosin.

## Data Availability

The data used to support the findings of this study are available from the corresponding author upon request.

## Additional Points

CT KUB was not done in all cases as a diagnostic modality due to financial reasons, and follow-up was up to three weeks as if we increase follow-up, then there may be more drop out cases and also patients in our region already presented late to hospital.
